# The Effect of a Booster Dose mRNA Vaccine on COVID-19 Infection in Kidney Transplant Recipients after Inactivated or Viral Vector Vaccine Immunization

**DOI:** 10.3390/vaccines10101690

**Published:** 2022-10-10

**Authors:** Sansanee Thotsiri, Rungtiwa Sittiudomsuk, Napun Sutharattanapong, Surasak Kantachuvesiri, Punlop Wiwattanathum

**Affiliations:** 1 Division of Nephrology, Department of Medicine, Faculty of Medicine Ramathibodi Hospital, Mahidol University, 270 Rama 6 Road, Ratchathewi, Bangkok 10400, Thailand; 2 Excellent Center for Organ Transplantation, Faculty of Medicine Ramathibodi Hospital, Mahidol University, Bangkok 10400, Thailand; 3 Department of Nursing, Faculty of Medicine Ramathibodi Hospital, Mahidol University, Bangkok 10400, Thailand

**Keywords:** inactivated vaccine, viral vector vaccine, mRNA vaccine, booster dose vaccine, SARS-CoV-2, COVID-19, kidney transplantation

## Abstract

The mortality rate after novel coronavirus infection, which causes severe acute respiratory distress syndrome (SARS-CoV-2), is much higher in kidney transplant recipients (KTRs) compared to the general population. Seroconversion after vaccination is also lower, and breakthrough infection is much higher. Many studies reported seroconversion rate after a booster (third) dose of vaccine but clinical outcomes received less attention. Here, we reported the impact of an mRNA vaccine booster dose on clinical outcomes of KTRs with SARS-CoV-2 infection. A total of 183 KTRs with SARS-CoV-2 infection were identified. Of 183 KTRs, 146 KTRs had sufficient data for analysis and were included in this study. Forty-eight patients (32.9%) received zero to 1 doses of vaccine (Group 1), thirty-one (21.2%) received two doses (Group 2), and sixty-seven (45.9%) received a booster dose (Group 3). Pneumonia developed in 50%, 23%, and 10% in Group 1, 2, and 3 (*p* < 0.001). Hospital admission requirement was 81%, 48%, and 12% (*p* < 0.001). Mortality rate was 26%, 3%, and 3% (*p* = 0.001). A multivariate analysis showed that only diabetes adversely affects mortality while the booster dose of the vaccine significantly reduced mortality. The booster dose of the vaccine is strongly recommended in all KTRs especially those with diabetes. Our study also suggested the timing of the booster dose vaccine to be administered within 4 months after the second dose.

## 1. Introduction

The worldwide healthcare system has been disrupted by the SARS-CoV-2 pandemic. The strategy was centered on vaccine research because of disease severity and the absence of effective treatments. Recent investigations in the general population have shown that vaccination can prevent SARS-CoV-2 infection as well as reduce the severity and mortality of the disease by generating humoral and cellular immune responses [1,2,3,4], however, the immune response in KTRs following vaccination is significantly weaker than in the immunocompetent population [5,6,7]. In terms of induced immunity by vaccination in KTRs, antibody seroconversion following inactivated vaccine was found to be insignificant [5,8,9]. In studies comparing viral vector and mRNA vaccines, the mRNA vaccination resulted in higher antibody levels than the viral vector vaccine [10]. Cell-mediated immune responses, on the other hand, can be achieved following any form of vaccine [11,12,13].

Initially, the recommendation was to receive two doses of the vaccine but studies in KTRs showed less than a 50% seroconversion rate [14,15,16]. Later studies found that antibody level decreased rapidly over time [17,18]. By 6 months, antibody level was too low to prevent infection and breakthrough infection can occur. KTRs can develop severe disease, with a 50–70% incidence of pneumonia and a 20–25% mortality rate during breakthrough infection [19,20,21]. A booster dose of the vaccination was used to increase antibody levels with the hope that it would reduce incidence and severity of breakthrough infection. Most studies found that a booster dose can significantly increase antibody levels, especially heterologous vaccine platform [22,23,24,25], but clinical outcomes were not reported. Almost all studies reported results of an mRNA vaccine. Data on using an mRNA vaccine as a booster dose in KTRs who received primary vaccination with inactivated and viral vector vaccines were scant.

In Thailand, the first available vaccine was an inactivated vaccine and later, a viral vector vaccine. Therefore, almost all KTRs initially received these two types of vaccines. When the mRNA vaccines became available, it was used as a booster dose in almost all KTRs. This study compares the clinical outcomes of KTRs who received a booster dose of an mRNA vaccine with those who did not receive the booster vaccine.

## 2. Methods

### 2.1. Study Design and Participants

This is a retrospective cohort study of KTRs diagnosed with SARS-CoV-2 infection at Ramathibodi Excellent Center for Organ Transplantation between January 2021 and July 2022. The inclusion criteria were the following: (1) adult recipients aged 18 and above who received either living (LRKT) or deceased donor kidney transplantation (DDKT); (2) received no vaccination or received one or two doses of an inactivated or viral vector vaccine; (3) if they had received a booster dose, it must be an mRNA vaccine. Patients who received other regimens of the vaccination such as the first or second dose of the vaccination with an mRNA vaccine, or did not receive a booster dose with an mRNA vaccine, were excluded. Patients with symptoms suggestive of SARS-CoV-2 infection or asymptomatic patients who had contact with SARS-CoV-2 cases were tested. The diagnosis of SARS-CoV-2 was made by using a real-time polymerase chain reaction (RT-PCR) or a rapid antigen test kit (ATK) (SARS-CoV-2). The study protocol was approved by the Institutional Human Research Ethics Committee of Mahidol University’s Faculty of Medicine Ramathibodi Hospital [MURA 2022/344]. The CoronaVac inactivated vaccine (Sinovac Biotech) and the ChAdOx1 nCoV-19 adenoviral vector vaccine (Vaxzevria, Oxford-AstraZeneca) were the first COVID-19 vaccines available in Thailand. Inactivated vaccines were administered four weeks apart [26], while viral vector vaccines were administered twelve weeks apart [27]. The third dose was a booster dose of an mRNA vaccine, either Pfizer BioNTech (BNT162b2) [28] or Moderna COVID-19 (mRNA-1273) vaccine [29] and was administered 4 to 6 months after the second dose according to the national public health policy. The patients were classified based on their vaccination status into 3 groups, with the first (Group 1) receiving either one dose of the inactivated/viral vector vaccine or were unvaccinated. The second group of patients (Group 2) received two doses of the inactivated/viral vector vaccine. Patients in the third group (Group 3) received the third dose of the vaccine as a booster dose with an mRNA vaccine.

### 2.2. Outcomes Measured

The primary goal of this study was to determine the effect of an mRNA vaccine booster dose on mortality reduction in SARS-CoV-2 infected KTRs. Secondary outcomes included the severity of COVID-19 disease, complications, the need for oxygen and ventilator support, hospital admission requirement, and the risk factors for death in KTRs.

### 2.3. Management during SARS-CoV-2 Infection

According to the regional standard of care for SARS-CoV-2 infection, patients were classified based on the severity and risk factors for severe disease. Those with severe disease were admitted to the hospital, while those with mild symptoms or who were asymptomatic were given self-care assignments at home or in hotel-based self-care assignments. Because of their immunosuppressive conditions, KTRs are categorized as having a high risk of developing a serious illness; thus, the majority of them received antiviral medication as soon as the diagnosis was confirmed. Anti-proliferative drugs were discontinued and calcineurin inhibitors (CNIs) were reduced to a low therapeutic range. Corticosteroids were continued or reintroduced if steroid withdrawal regimens were used. For patients who required steroid treatment, methylprednisolone or dexamethasone were used and prednisolone was discontinued.

### 2.4. Statistical Analysis

For categorical data, baseline characteristics were expressed as frequency (percentage). For the group comparison, the Chi-square test was used. Continuous measurements were reported as mean and standard deviation (SD) for normal distributions and median and interquartile range (IQR) for non-normal distributions. A one-way ANOVA and the Kruskal–Wallis H test were used to compare their differences [30].

The primary objective was to assess the difference in mortality, which was assessed using a logistic regression analysis as a relative risk ratio (RR) of death between groups with a 95% confidence interval (CI), and was also reported in the Kaplan–Meier time-to-event analysis using the log-rank test [31]. Secondary outcomes were also reported as a relative risk ratio with 95% CI. To identify factors that may affect mortality (vaccination status, recipient age of 60 years or older, type of donor, diabetes, and obesity), univariate and multivariate logistic regression analyses were used. All statistical analyses were carried out using IBM SPSS version 23 software.

## 3. Results

### 3.1. Patients

There were 183 kidney transplant recipients with confirmed SARS-CoV-2 infection during the study period. One hundred and forty-six patients were included and thirty-seven patients were excluded (initial vaccination with mRNA vaccine, *n* = 13, received second dose with mRNA vaccine, *n* = 14, received non-mRNA vaccine for booster dose, *n* = 10). There were 48 (32.9%) patients in Group 1, 31 (21.2%) patients in Group 2, and 67 (45.9%) patients in Group 3. The mean age of the patients was 47 years old, with 56% of them being male. Ninety-six patients (66%) received a kidney from deceased donors, while fifty (34%) received a kidney from living donors. The average time after transplant was 46 months. Comorbidities included hypertension (62%), diabetes (20%), ischemic heart disease (6%), and obesity (14%). The baseline characteristics are shown in Table 1.

In Group 1 (*n* = 48), 29 (60%) were unvaccinated, 17 (36%) received one dose of the ChAdOx1 nCoV-19 vaccine, and 2 (4%) received one dose of the CoronaVac. In Group 2 (*n* = 31), 19 (61%) patients received two doses of the ChAdOx1 nCoV-19 vaccine, 7 (23%) received two doses of the CoronaVac, and 5 (16%) received a first dose of the CoronaVac followed by the ChAdOx1 nCoV-19 vaccine as a second dose. In Group 3 (*n* = 67), 50 (75%) received two doses of the ChAdOx1 nCoV-19 vaccine, 8 (12%) received two doses of the CoronaVac, and 9 (13%) received the CoronaVac as a first dose and the ChAdOx1 nCoV-19 vaccine as a second dose. The median time to receiving a booster dose was 116 days after the second dose and the median time since the last vaccination to diagnosis of SARS-CoV-2 infection was 32, 137, and 88 days in Group 1, 2 and 3, respectively.

### 3.2. Hospital Course

Antiviral regimens were used as follows: Favipiravir in 145 patients (79%), Molnupiravir in 4 patients (3%), and Remdesivir in 5 patients (4%), which were not different between groups. For COVID-19 treatment, 10% required corticosteroids (either methylprednisolone or dexamethasone). Three patients required biologic immunomodulatory agents and one patient required hemoperfusion. Immunosuppressive management was the same in all groups, with the majority of patients continuing CNIs and discontinuing mycophenolate.

### 3.3. Clinical Outcomes

**Figure 1:**
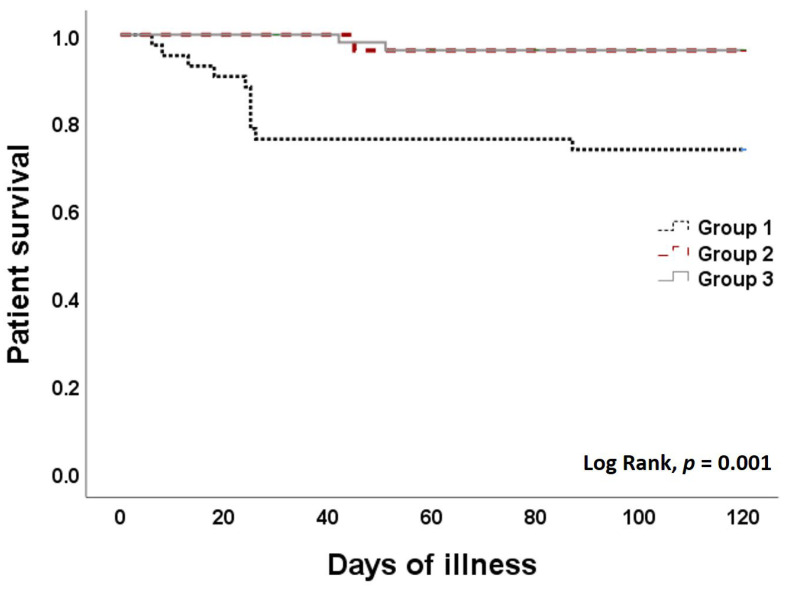
Kaplan–Meier curve of survival in kidney transplant recipients with SARS-CoV-2 infection. Group 1 (black dash line), Group 2 (red dash line) and Group 3 (grey solid line).

The overall mortality rate was 10% (26% vs. 3% vs. 3% in Group 1, Group 2, and Group 3, respectively, *p* = 0.001). The log rank test showed separation of the survival curves for patients in Groups 1 vs. 2 and 3, with a *p*-value of 0.001 (Figure 1). Pneumonia was found in 26% of patients and was substantially more prevalent in Group 1 compared to Group 2 and 3 (50% vs. 23% vs. 10%, respectively, *p* = 0.001). Invasive mechanical ventilation was used in 6% of patients (15%, 0%, and 2% in Group 1, 2, and 3, respectively). Twelve patients (25%) in Group 1, four patients (13%) in Group 2, and three patients (5%) in Group 3 required oxygen supplement (*p* = 0.001). Hospital admission was required in 81% vs. 48% vs. 12% (*p* < 0.001) (Table 2). A subgroup analysis of Group 3 in patients who received two doses of the inactivated vaccine (*n* = 17) vs. two doses of the viral vector vaccine (*n* = 50) did not show a statistical difference (see Supplementary Materials Table S1). 

A univariate analysis revealed that diabetes, recipients aged 60 years or older, and vaccination status affect mortality. However, a multivariate analysis revealed that diabetes significantly increased mortality (odds ratio 7.411; 95% CI, 1.83–29.95; *p* = 0.005) while receiving three doses of vaccines (Group 3) significantly decreased mortality (odds ratio 0.184; 95% CI, 0.03–0.99; *p* = 0.049). (Table 3).

## 4. Discussion

Our study demonstrated the beneficial effect of an mRNA booster dose vaccination on reducing pneumonia, oxygen requirement, hospitalization, and death in KTRs infected with SARS-CoV-2. In comparison to patients who received a booster mRNA vaccine, the rate of pneumonia doubled in patients who received two doses of the vaccine (either inactivated or viral vector vaccine) and five-fold in patients who received only one dose of a vaccine or no vaccine at all. Patients who received a booster mRNA vaccine had lower oxygen requirements, fewer required hospital admission. And most importantly, the booster mRNA group had the lowest mortality rate. In our study, there was no difference in antiviral medication administration in each group, which underscores the beneficial effect of vaccination.

SARS-CoV-2 infection had a very high mortality rate among KTRs, particularly those who did not receive any vaccines. Previous studies reported a mortality rate of 20–37% in KTRs who did not receive any vaccines [21,32,33]. Two doses of vaccination in KTRs reduced the mortality rate to 10–20% [19,21,34], however, there was some breakthrough infection in this setting and the rate of pneumonia were still high, at 50–70% [19,21,34,35]. Breakthrough infection rates in KTRs receiving two doses of vaccination were reported to be around 0.4 to 1.5% [34,36], but this incidence was 20 to 80 times higher than that in the general population [37]. The outcome following breakthrough infection was poor, with around a 10 to 20% mortality rate [19,21,34].

Reasons of breakthrough infection were as follows: first, when compared to the general population, KTRs had a much lower seroconversion rate of only 20% (5) and 40–50% [14,15,16] after two doses of the inactivated vaccine and an mRNA vaccine, respectively; second, because of the antibody induced by the first two doses of vaccination, it decreased rapidly. By 3 to 6 months after the last dose, the antibody level was too low to be protective resulting in a decrease in level of protection over time [17,18]. The booster dose vaccination strategy was implemented in the hope of reducing disease severity and mortality.

There were two strategies for using the booster dose vaccine: homologous (using the same vaccine platform) and heterologous (using a different vaccine platform) when compared to the previous two doses of the vaccination. Several studies have found that heterologous vaccines outperform homologous vaccines in terms of higher antibody production and T-cell activation in both the general population and KTRs [22,23,38]. The rate of seroconversion can be achieved in 60–70% of primary non-responders with a heterologous booster dose, leaving 20–25% of KTRs with no seroconversion after receiving a booster dose [15,16,39]. However, there was no mention of clinical outcomes of KTRs infected with SARS-CoV-2 following a booster dose.

There were some disagreements on the role of a booster dose whether it was necessary [40,41]. Our findings demonstrated that using a booster dose vaccine significantly reduced disease severity, hospitalization, and mortality. The multivariate analysis showed that diabetes is a significant factor for mortality, so KTRs with diabetes should receive special attention and priority in receiving a vaccination. In our opinion, the primary course of vaccination in KTRs and other immunocompromised state should comprise three doses, not only two doses of the vaccine. Based on our data, the booster dose was administered at the median of 111 days and SARS-CoV-2 infection occurred 137 days after the second dose of the vaccine. With this information, the third dose of the vaccine should be administered within 4 months after the second dose to prevent infection after two doses of vaccine. Should KTRs get an infection later on, they are better protected with three doses of the vaccine with a significantly less severe disease and lower mortality.

A limitation of our study was that vaccination regimens in Thailand were heterogeneous, depending on the availability of vaccines. A comparison of effectiveness of different regimens in preventing infection or reducing disease severity was difficult. It is impossible to identify the “best” regimen of vaccination for KTRs. However, our data clearly showed that the third dose of the vaccine is beneficial and every KTR should be encouraged to receive at least the third dose of vaccine.

## 5. Conclusions

Our study demonstrated that the third (booster) dose of the vaccine significantly reduced disease severity and mortality in KTRs infected with SARS-CoV-2. Every KTR should be encouraged to receive the third dose of vaccine and the third dose should be administered within 4 months after the second dose.

## Figures and Tables

**Table 1:** Baseline characteristics.

	Total *n* = 146	Group 1 *n* = 48	Group 2 *n* = 31	Group 3 *n* = 67	*p*-Value
Age (y), mean (SD)	47 (12)	49 (13)	46 (11)	46 (12)	0.472
Age ≥ 60, *n* (%)	24 (16)	11 (23)	3 (10)	10 (15)	0.246
Gender, *n* (%) Male Female	81 (56) 64 (44)	31 (65) 17 (35)	12 (39) 19 (61)	38 (57) 29 (43)	0.075
BMI (kg/m^2^), mean (SD)	24.0 (5.2)	24.4 (5.4)	24.1 (5.2)	23.7 (5.1)	0.771
Obesity (BMI ≥ 30), *n* (%)	20 (14)	8 (17)	4 (13)	8 (12)	0.760
Kidney transplant status Deceased donor transplantation, *n* (%) Living relate transplantation, *n* (%)	96 (66) 50 (34)	28 (58) 20 (42)	24 (77) 7 (23)	44 (66) 23 (34)	0.196
Post-transplant time (months), median (IQR)	46 (24,86)	55 (27,108)	34 (15,68)	43 (21,85)	0.224
Immunosuppressive regimens, *n* (%) Calcineurin inhibitors Mycophenolate mTor inhibitios Steroids	144 (99) 141 (97) 2 (1) 144 (99)	46 (96) 44 (92) 2 (5) 47 (98)	31 (100) 31 (100) 0 30 (97)	67 (100) 66 (98) 0 67 (100)	0.126 0.069 0.126 0.386
Comorbidity, *n* (%) Diabetes Hypertension Ischemic heart disease	28 (20) 90 (62) 8 (6)	15 (36) 29 (69) 5 (12)	6 (19) 15 (48) 0	7 (10) 46 (69) 3 (5)	0.037 0.216 0.175
Interval after last vaccine (d), median (IQR)		32 (14,58)	137 (68,191)	88 (54,142)	

mTOR, mammalian target of rapamycin.

**Table 2:** Outcomes of SARS-CoV-2 infection in kidney transplant recipients.

Outcomes, *n* (%)	Total *n* = 146	Group 1 *n* = 48	Group 2 *n* = 31	Group 3 *n* = 67	*p*-Value
Death	14 (10)	11 (26)	1 (3)	2 (3)	0.001
Pneumonia	38 (26)	24 (50)	7 (23)	7 (10)	<0.001
Oxygen requirement					<0.001
Invasive mechanical ventilation	8 (6)	7 (15)	0	1 (2)	
High flow nasal cannula	4 (3)	3 (6)	1 (3)	0	
Nasal cannula	15 (10)	9 (19)	3 (10)	3 (5)	
No requirement	119 (82)	29 (60)	27 (87)	63 (94)	
Hospital admission requirement	57 (41)	34 (81)	15 (48)	8 (12)	<0.001

**Table 3:** Univariate and multivariate analysis of factors-associated mortality in SARS-CoV-2 infected kidney transplant recipients.

Factors	Univariate Analysis		Multivariate Analysis	
	OR (95% CI)	*p*-Value	OR (95% CI)	*p*-Value
Vaccination group Group 1 Group 2 Group 3	Ref 0.112 (0.014–0.918) 0.103(0.022–0.492)	0.041 0.004	0.162 (0.018–1.496) 0.184 (0.034–0.994)	0.109 0.049
Recipient age ≥ 60 years	4.750 (1.475–15.296)	0.009	3.092 (0.743–12.862)	0.121
Diabetes	13.382 (3.743–47.846)	<0.001	7.411 (1.833–29.957)	0.005
Obesity (BMI ≥ 30)	2.900 (0.813–10.346)	0.101	1.871 (0.376–9.313)	0.444
Deceased donor transplantation	1.337 (0.397–4.500)	0.639		

CI, confidence interval; OR, odds ratio.

## Data Availability

Data are available upon request.

## Supplementary Materials

The following supporting information can be downloaded at: https://www.mdpi.com/article/10.3390/vaccines10101690/s1, Table S1: title Subgroup analysis of group 3 patient who received inactivated VS. viral vector vaccine.

## Abbreviations

CI	confidence interval
CNIs	calcineurin inhibitors
COVID-19	coronavirus virus disease 2019
DDKT	deceased donor kidney transplantation
IQR	interquartile range
KTRs	kidney transplant recipients
LRKT	living donor kidney transplantation
mRNA	messenger ribonucleic acid
mTOR	mammalian target of rapamycin
RR	relative risk ratio
RT-PCR	real-time polymerase chain reaction
SARS-CoV-2	severe acute respiratory syndrome coronavirus 2
SD	standard deviation
