# Supplementary material for: The Effect of a Booster Dose mRNA Vaccine on COVID-19 Infection in Kidney Transplant Recipients after Inactivated or Viral Vector Vaccine Immunization
Source: Vaccines (Basel). 2022 Oct 10;10(10):1690. doi: 10.3390/vaccines10101690 (PMC9611339; doi:10.3390/vaccines10101690)
Supplement: Supplementary file 1 [file vaccines-10-01690-s001.zip › vaccines-1905584-supplementary.pdf]

Supplementary

Table S1. Subgroup analysis of group 3 patient who received inactivated VS. viral vector vaccine

Outcomes, n (%)	2 viral vector vaccine + mRAN N = 50	2 inactivated vaccine + mRAN N = 17	*p*-value
**Death**	2 (4)	0 (0)	0.178
**Pneumonia**	7 (14)	0 (0)	1.000
**Oxygen requirement**			0.485
Invasive mechanical ventilation	1(2)	0	
High flow nasal cannula	0	0 (0)	
Nasal cannula	3 (6)	0 (0)	
No requirement	46 (92)	17 (100)	
**Hospital admission requirement**	9 (18)	1 (6)	0.312
